# A Sea-Sky Line Detection Method for Unmanned Surface Vehicles Based on Gradient Saliency

**DOI:** 10.3390/s16040543

**Published:** 2016-04-15

**Authors:** Bo Wang, Yumin Su, Lei Wan

**Affiliations:** National Key Laboratory of Science and Technology on Underwater Vehicle, Harbin Engineering University, Harbin 150001, China; suyumin@hrbeu.edu.cn (Y.S.); wanlei@hrbeu.edu.cn (L.W.)

**Keywords:** unmanned surface vehicle, sea-sky line, gradient saliency, region growing, line support region

## Abstract

Special features in real marine environments such as cloud clutter, sea glint and weather conditions always result in various kinds of interference in optical images, which make it very difficult for unmanned surface vehicles (USVs) to detect the sea-sky line (SSL) accurately. To solve this problem a saliency-based SSL detection method is proposed. Through the computation of gradient saliency the line features of SSL are enhanced effectively, while other interference factors are relatively suppressed, and line support regions are obtained by a region growing method on gradient orientation. The SSL identification is achieved according to region contrast, line segment length and orientation features, and optimal state estimation of SSL detection is implemented by introducing a cubature Kalman filter (CKF). In the end, the proposed method is tested on a benchmark dataset from the “XL” USV in a real marine environment, and the experimental results demonstrate that the proposed method is significantly superior to other state-of-the-art methods in terms of accuracy rate and real-time performance, and its accuracy and stability are effectively improved by the CKF.

## 1. Introduction

In recent years, with their rapid development USVs are playing more and more important roles in various areas such as meteorological monitoring, maritime search and rescue, enemy reconnaissance and precision military strikes. To navigate autonomously and accomplish a variety of missions without human interventions, USVs need to be equipped with different sensors like radars, cameras and thermal infrared imagers to perceive and comprehend the marine environment and all kinds of targets around them, and intelligent behaviors including target detection, identification and tracking are implemented autonomously. As a result, cameras have become an indispensable important sensor for USVs due to their high resolution, abundant information, similarity to the human visual system and low cost.

In the optical images obtained by cameras in the marine environment, the sea-sky line (SSL) is one of the most important cues. Firstly, in optical images where the SSL represents a dividing line, the sky region above and the sea region below have different pixel value distributions [1], so the accurate detection of SSL is of great benefit to target detection. Secondly, while a distant target enters into the field of view (FOV) of a camera, in optical images it always appears around the SSL, and then moves into the sky region or the sea region during the approaching process, therefore the detection of SSL is an effective measure to improve the target detection, identification and tracking performance through narrowing the target searching range and suppressing false detections. Thirdly, according to the position and motion pattern of the detected SSL, the motion status of USVs can be estimated and motion compensation of images can be implemented, which is quite applicable to USV target detection and tracking.

In optical images the SSL presents itself a dividing line composed of a gradient of maximum pixels between the sky region and the sea region, which is a straight line without consideration of sea surface curvature and optical distortion. However, in optical images from real marine environments there often exist strong interferences, including cloud clutter and sea glint, besides, changeable weather conditions like fog, snow or rain can seriously decrease the image contrast and sharpness and brings about noise in images, causing great difficulties for accurate SSL detection.

Kim, *et al.* extracted horizon pixels based on calculation of a column directional gradient, then a random sample consensus (RANSAC) algorithm was applied to select inlier horizon pixels and the final horizon was detected stably by least squares optimization [2]. However, the RANSAC line fitting method is quite sensitive to widely distributed noise and strong edges and the authors claimed sensor pose information was exploited to predict the horizon location.

Zou, *et al.* proposed a shearlet-based edge identification method for SSL detection in infrared images [3]. Shearlets are capable of direction information analysis and can provide edge geometric features, but the computational complexity is rather high and such a method is not suitable for real-time applications at all.

Rahman, *et al.* accomplished horizon detection with the Canny edge detection and Hough transform methods [4,5,6,7,8], but the Hough transform needs a compromise between detection accuracy and computational complexity, moreover, it suffers from interference of strong edges and noise like cloud clutter and wave glint, and the Hough transform often fabricates false line segments.

Tang, *et al.* proposed a SSL detection method based on Radon transform [9], but this method faces the same problems as the Hough transform, besides, the Radon transform cannot determine the endpoints of line segments.

Rahul, *et al.* proposed a theoretical framework for generating pseudospectral images from spectrum analysis of color images, and then an ellipse fitting method derived from calculation of inertia moments of connected components in binary edge images was introduced for horizon detection [10]. However, when the image contrast or sharpness is weak, or strong interference edges exist, the probability of false detection increases significantly.

Ahmad, *et al.* designed a maximally stable external edge detection method on the basis of Canny edges, then a support vector machine classifier was trained to classify edge points using local scale invariant features, and finally, a dynamic programming method was applied to extract the horizon lines [11]. However, machine learning methods always need a large amount of samples to train the classifier, and the great variations of illumination, reflection, scattering and clutter in marine environments brings great challenges for these methods.

Nasim, *et al.* presented an approach employing the segmentation of sea surface scenes into several clusters with a K-means algorithm, then analyzed image clusters to extract the sky region and find a horizon path between the sky region and the other clusters [12], but for these region segmentation methods, special features in the sea-sky scene such as low contrast, weak sharpness, cloud clutter and sea glint may lead to large misalignment or false horizon line detections.

In this paper a novel saliency-based SSL detection method is proposed. Through the computation of gradient saliency the line features of SSL are enhanced effectively, while other interference factors are relatively suppressed, and line support regions (LSR) are obtained by a region growing method based on gradient orientation. The SSL identification is achieved according to region contrast, line segment length and orientation features of LSRs, and an optimal state estimation of SSL detection is implemented by introducing CKF.

The structure of this paper is as follows: firstly, the hardware architechture and the principle of the optoelectronic imaging unit mounted on the “XL” USV are introduced. Then the key algorithms, such as gradient saliency calculation, region growing algorithm based on gradient orientation, improvement of detected line features, identification of SSL, and improvement of accuracy and stability based on CKF, are detailed in the following sections. Finally, our proposed method is tested on a benchmark dataset from the “XL” USV in a real marine environment to demonstrate its effectiveness.

## 2. Hardware Architecture

**Figure 1:**
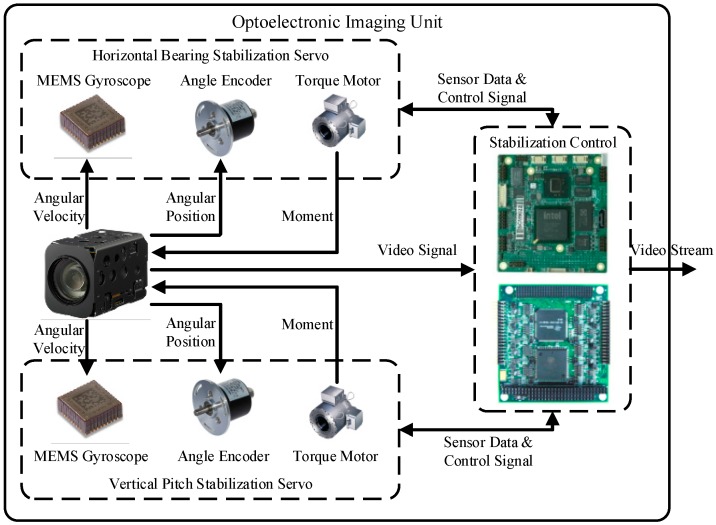
Hardware architecture of the optoelectronic imaging unit.

An optoelectronic imaging unit capable of 2-axis image stabilization is developed in our research work, and it is mounted on the “XL” USV to acquire optical images in real marine environments. The hardware architecture is presented in Figure 1, where the optoelectronic imaging unit consists of three main parts: horizontal bearing stabilization servo, vertical pitch stabilization servo and stabilization control. Horizontal bearing stabilization servo, the principle of which is the same as vertical pitch stabilization servo, uses a MEMS gyroscope to measurethe horizontal angular velocity caused by USV motion disturbances on the camera, and uses an angle encoder to measure the horizontal angular position of the camera. The sensor data is transmitted to the stabilization control, which generates control signals for the torque motor according to PID control law, and the torque motor drives the slip ring on which the camera is mounted to rotate to compensate the horizontal angular velocity caused by disturbances.

The digital video signal of the camera is grabbed and compressed into a video stream by stabilization control system, which executes some intelligent actions such as SSL detection, target detection, target identification and target tracking at the same time. The video stream can be saved on local hard disks or transmitted to a real-time monitoring terminal far away through a suitable datalink.

## 3. Detection of Line Features

**Figure 2:**
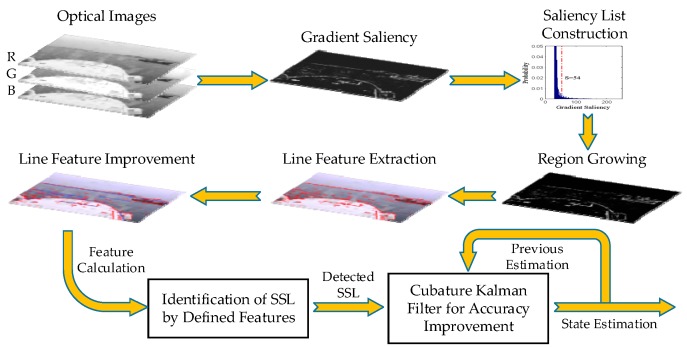
Block diagram of the proposed SSL detection method.

The diagram of the proposed SSL detection method is presented in Figure 2. Firstly, the gradient saliency is calculated based on RGB color space of optical images. Secondly, the saliency list is constructed and a region growing algorithm is applied to produce LSRs. Thirdly, the line features are extracted and improved on the basis of detected LSRs. Finally, the real SSL needs to be identified from candidate line features, and the accuracy is further improved by CKF according to previous state estimation and current detection.

### 3.1. Gradient Saliency

Saliency originates from visual uniqueness, unpredictability, rarity or surprise, and it is tightly related to human perception and processing of visual stimuli. The human visual system always pays more attention to variations in images like color, gradient and edges, and high gradient edges arouse intense stimuli in the visual system, in other words, high gradient edges obtain high saliency [13]. In this paper global gradient saliency based on the RGB color space is introduced. The reason for choosing RGB color space instead of gray space in the calculation of gradient saliency is that gradient information is lost in the transformation from a RGB color image to a gray image, for instance, different color values could be projected into the same gray value [14], which will have a negative influence on SSL detection as a result.

Given an optical image *I*, the gradient submatrix for each color can be calculated through convolution of the color value submatrix with Sobel operators, thus the gradient saliency of a pixel *i* in image *I* is formulated as a distance measure between the gradient of pixel *i* and the other pixels:

(1) $$S(i)=\sum_{j\in I}D(g_{i},g_{j})$$

where $D(g_{i},g_{j})$ denotes the distance measured between gradient vectors $g_{i}$ and $g_{j}$ of pixels *i* and *j* in image *I*. Let the pixel number in image *I* be *N* the computational complexity of gradient saliency calculation for all pixels is *O*(*N*^2^). Actually, the definition of gradient saliency ignores spatial relations among pixels, therefore pixels with the same gradient will have the same gradient saliency, and gradient saliency can be rewritten as follows [13]:

(2) $$S(i)=\sum_{k=1}^{n}h(g_{k})D(g_{i},g_{k})$$

where *n* is the number of distinct gradient vectors in image *I*, $g_{k}$ and $h(g_{k})$ denote the gradient vector and its probability, respectively. Then the computational complexity of gradient saliency calculation is reduced to *O*(*N + n*^2^). The distance measure $D(g_{i},g_{k})$ is described as follows:

(3) $$\begin{array}{l} D(g_{i},g_{k})={\left\|g_{i}-g_{k}\right\|}_{1}\\ g_{i}={\left[\begin{array}{ccc} \partial R_{i} & \partial G_{i} & \partial B_{i} \end{array}\right]}^{T} \end{array}$$

where ${\left\|g_{i}-g_{k}\right\|}_{1}$ denotes the $\ell_{1}$ norm of vector $g_{i}-g_{k}$. If the gradient level of each color is normalized to *l*, then the number of distinct gradients is *n* = *l*^3^ and there will be 3*l* kinds of gradient saliency. The accurate quantization of gradient saliency is beneficial to SSL detection accuracy, but the computational cost is high and there will be more SSL gaps. Subsequently, in this paper the gradient amplitude and orientation are used for gradient saliency calculation as follows [15]:

(4) $$\begin{array}{l} g_{i}={\left\{\frac{1}{2}\left[(\varphi_{xx}+\varphi_{yy})+(\varphi_{xx}-\varphi_{yy})\cdot cos2\theta_{i}+2\varphi_{xy}\cdot \sin2\theta_{i}\right]\right\}}^{\frac{1}{2}}\\ \theta_{i}=\frac{1}{2}\arctan\left(\frac{2\varphi_{xy}}{\varphi_{xx}-\varphi_{yy}}\right) \end{array}$$

where $\theta_{i}$ is gradient orientation of pixel *i*, and quantities $\varphi_{xx}$, $\varphi_{xy}$ and $\varphi_{yy}$ are defined as follows:

(5) $$\begin{array}{l} \varphi_{xx}=\frac{\partial R_{i}}{\partial x}\frac{\partial R_{i}}{\partial x}+\frac{\partial G_{i}}{\partial x}\frac{\partial G_{i}}{\partial x}+\frac{\partial B_{i}}{\partial x}\frac{\partial B_{i}}{\partial x}\\ \varphi_{xy}=\frac{\partial R_{i}}{\partial x}\frac{\partial R_{i}}{\partial y}+\frac{\partial G_{i}}{\partial x}\frac{\partial G_{i}}{\partial y}+\frac{\partial B_{i}}{\partial x}\frac{\partial B_{i}}{\partial y}\\ \varphi_{yy}=\frac{\partial R_{i}}{\partial y}\frac{\partial R_{i}}{\partial y}+\frac{\partial G_{i}}{\partial y}\frac{\partial G_{i}}{\partial y}+\frac{\partial B_{i}}{\partial y}\frac{\partial B_{i}}{\partial y} \end{array}$$

Then the distance measure $D(g_{i},g_{k})$ is simplified as follows:

(6) $$D(g_{i},g_{k})=\left|g_{i}-g_{k}\right|$$

If the gradient level is normalized to *l*, then the number of distinct gradients is *n = l* and there will be *l* kinds of gradient saliency. The computational cost is effectively reduced, and experiments show that the continuity and accuracy of detected SSL are satisfactory.

**Figure 3:**
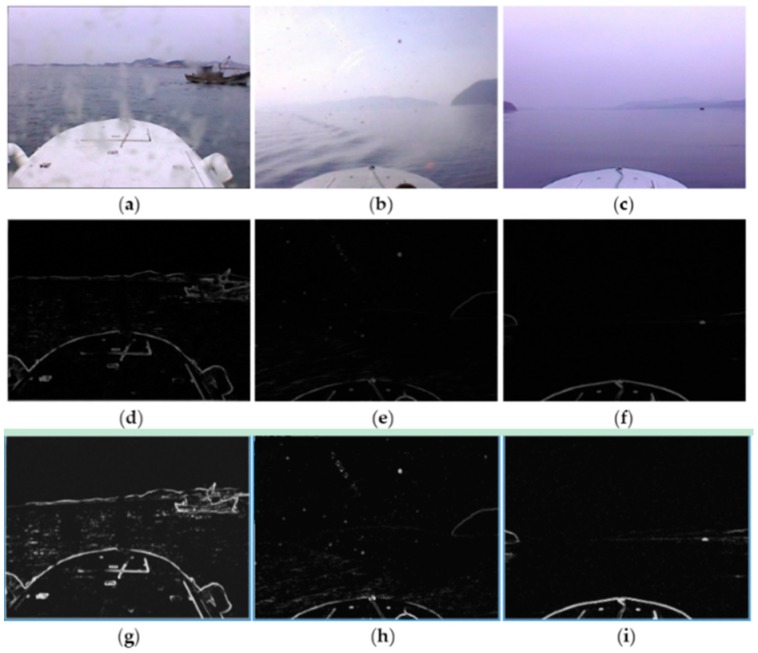
Gradient maps and gradient saliency maps of optical images in typical adverse weather conditions. (**a**–**c**) are original images; (**d**–**f**) are gradient maps; (**g**–**i**) are gradient saliency maps.

The gradient maps and gradient saliency maps of optical images acquired by the “XL” USV in typical adverse weather are presented in Figure 3. Figure 3a–c shows the typical original images obtained in rainy weather, sunny weather with strong illumination and foggy weather, respectively.

The gradient maps shown in Figure 3d–f are obtained through convolution of the original images with Sobel operators; note that there exist high gradient edges formed by certain elements such as the USV hull, mountains, sunlight illumination and wave glint, which make it very difficult to distinguish and accurately detect SSLs with relatively weak gradient. In gradient saliency maps, as shown in Figure 3g–i, the line features of SSL are effectively enhanced, although strong edges formed by various interference still exist and part of the SSL is missing, the SSL can already be detected accurately in all probability.

### 3.2. Region Growing Based on Gradient Orientation

The basic idea of region growing methods is that spatially neighboring pixels with similar properties should be clustered together to constitute connected regions. The SSL in optical images shows typical line features, which are actually rectangular regions with a width of several pixels formed by neighboring pixel sets with high gradient and similar orientation, therefore we can consider the use of region growing methods to detect line features in gradient saliency maps [16]. In this paper the seed points of region growing are selected according to gradient saliency, the criterion for growth is defined as similarity of gradient orientation, and the proximate rectangle regions with similar gradient orientation, known as LSR, are obtained as a result. Observing gradient saliency maps, we can conclude that pixels with high gradient saliency and geometric property actually account for a very small proportion, thus we can select a specific proportion of pixels with the highest gradient saliency to participate in region growing, and that will effectively decrease the computational complexity of the region growing method. The region growing process based on gradient orientation can be described as follows:

*Step 1.* Calculate the histogram of gradient saliency, select 10% of pixels with the highest gradient saliency in the histogram and sort them in the order of gradient saliency to construct a saliency list *L*, set all the pixels in *L* as “unlabeled”;

*Step 2.* Pick up an “unlabeled” pixel *i* from saliency list *L* in sequence, initialize a LSR *C_k_* as a null set, add pixel *i* into *C_k_* and set it as “labeled” in *L*, and initialize the region orientation $\theta_{k}$ of *C_k_* as gradient orientation of pixel *i*;

*Step 3.* For each pixel *j* in *C_k_*, if its 8-connected pixel *l* is “unlabeled” in saliency list *L*, and satisfies the condition as follows [16]:

(7) $$\left|\theta_{k}-\theta_{l}\right|<\tau$$

where $\theta_{l}$ is gradient orientation of pixel *l*, τ is tolerance of region growing and τ=π/8, then add pixel *l* into *C_k_* and set it as “labeled”. Update the region orientation as follows:

(8) $$\theta_{k}=\arctan\frac{\sum_{j\in C_{k}}\sin\theta_{j}}{\sum_{j\in C_{k}}\cos\theta_{j}}$$

If there is a new pixel added into *C_k_*, then repeat this step; 

*Step 4*. Repeat Steps 2 and 3 until all the pixels in saliency list *L* are “labeled”.

**Figure 4:**
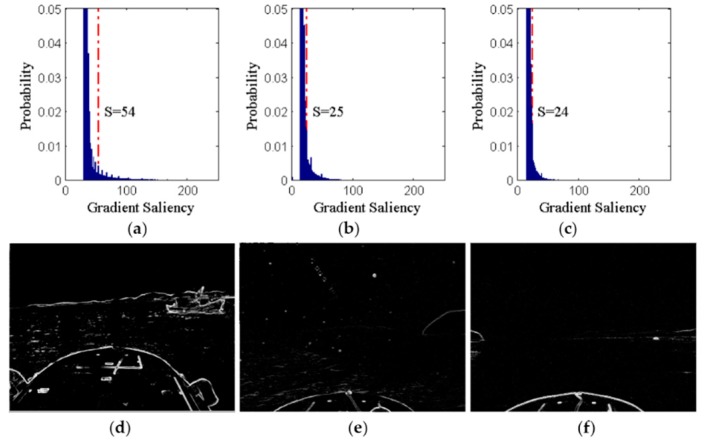
Histograms of gradient saliency and saliency list images. (**a**–**c**) are histograms of gradient saliency; (**d**–**f**) are saliency list images.

As shown in Figure 4a–c, the gradient saliency histograms are calculated by the gradient saliency maps shown in Figure 3g–i, where the red dot dashed lines denote the thresholds of 10% of pixels with the highest gradient saliency. Figure 4d–f are saliency lists displayed in graphical format showing that the saliency lists essentially contain all the effective edges in the corresponding gradient saliency maps.

**Figure 5:**
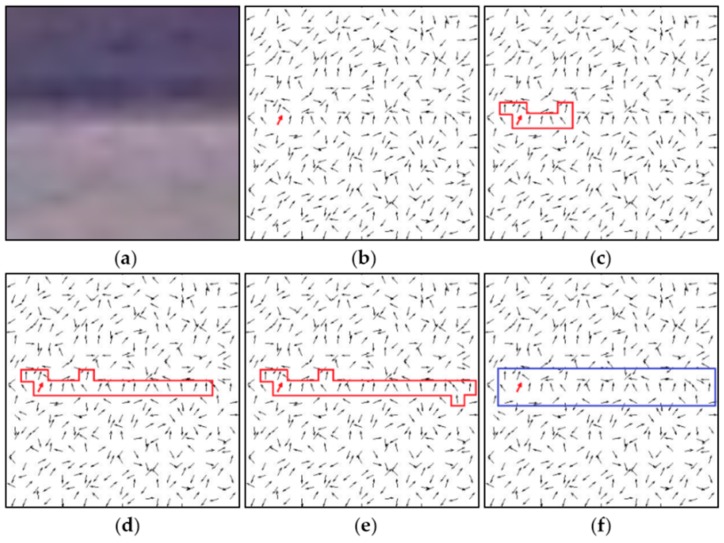
Region growing process based on gradient orientation. (**a**) is the original image of the local region; (**b**–**d**) show the LSR in growing; (**e**) is the final LSR in the end of region growing; (**f**) is minimum enclosing rectangle of the LSR.

The region growing process based on gradient orientation is illustrated by the example of a 20 × 20 local region around SSL, as shown in Figure 5.

Figure 5a presents the original image of the local region, and the gradient orientation of each pixel is indicated by an arrow, as depicted in Figure 5b, where the red one denotes a seed point with maximum gradient saliency. In Figure 5c a LSR is obtained by region growing from the seed point, through appending to the seed point neighboring pixels that have high gradient saliency and similar gradient orientation, the LSR continues growing along the SSL, as shown in Figure 5c, until the final LSR depicted in Figure 5e is formed. The blue rectangle in Figure 5f is the minimum enclosing rectangle of the obtained LSR.

### 3.3. Line Feature Extraction and Improvement

LSRs obtained by the region growing method indicate line features that exist in optical images, the mathematical description of line features can be generated by calculating statistical parameters of the LSR. The saliency centroid $\left({\bar{x}}_{k},{\bar{y}}_{k}\right)$ of LSR *C_k_* can be calculated as follows:

(9) $${\bar{x}}_{k}=\frac{\sum_{i\in C_{k}}S(i)x_{i}}{\sum_{i\in C_{k}}S(i)},{\bar{y}}_{k}=\frac{\sum_{i\in C_{k}}S(i)y_{i}}{\sum_{i\in C_{k}}S(i)}$$

where $\left(x_{i},y_{i}\right)$ is pixel coordinates of pixel *i*, S(i) is gradient saliency of pixel *i*. The correlation matrix $\Phi_{k}$ of LSR *C_k_* is formulated as follows [16]:

(10) $$\Phi_{k}=\left[\begin{array}{cc} \phi_{xx} & \phi_{xy}\\ \phi_{xy} & \phi_{yy} \end{array}\right]$$

where $\phi_{xx}$, $\phi_{xy}$ and $\phi_{yy}$ are second order saliency central moments defined as follows:

(11) $$\begin{array}{l} \phi_{xx}=\frac{\sum_{i\in C_{k}}S(i){\left(x_{i}-{\bar{x}}_{k}\right)}^{2}}{\sum_{i\in C_{k}}S(i)},\phi_{yy}=\frac{\sum_{i\in C_{k}}S(i){\left(y_{i}-{\bar{y}}_{k}\right)}^{2}}{\sum_{i\in C_{k}}S(i)}\\ \phi_{xy}=\frac{\sum_{i\in C_{k}}S(i)(x_{i}-{\bar{x}}_{k})(y_{i}-{\bar{y}}_{k})}{\sum_{i\in C_{k}}S(i)} \end{array}$$

The main orientation ${\bar{\theta }}_{k}$ of LSR $C_{k}$ should be the angle denoted by eigenvector associated with the smaller eigenvalue of correlation matrix $\Phi_{k}$. The line feature represented by $C_{k}$ corresponds to a geometric object that is a minimum enclosing rectangle $R_{k}$ of $C_{k}$ with the main orientation ${\bar{\theta }}_{k}$. To calculate the length $l_{k}$ and width $w_{k}$ of $R_{k}$ for LSR $C_{k}$, which are also the size of the line feature represented by $C_{k}$, all the pixels in $C_{k}$ are rotated by ${\bar{\theta }}_{k}$ around centroid $\left({\bar{x}}_{k},{\bar{y}}_{k}\right)$, and the length $l_{k}$ and width $w_{k}$ are set to the smallest values that make the rectangle cover the complete LSR $C_{k}$.

**Figure 6:**
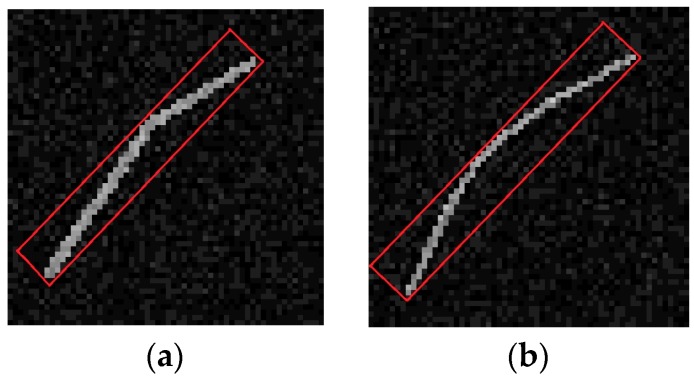
False detections of line features. (**a**) is polyline edge; (**b**) is curve edge.

The region growing method exploits similarity of gradient orientation as the predefined criterion for growth, the neighboring pixels, the gradient orientation of which is within the tolerance to main orientation of LSR, are appended to the LSR, thus some curve edges with small curvature or polyline edges with small orientation change may grow into LSR. In two local regions of the gradient saliency maps shown in Figure 6, due to the small variation of gradient orientation, the polyline edge in Figure 6a and the arc edge in Figure 6b, which are marked by red rectangles, form two false LSRs after the region growing process. If statistical parameters are computed on the basis of a false LSR, the line feature error will be huge, thus the curve edges and polyline edges should be approximately interpreted as several line features.

A LSR is improved according to its aligned point density, which is defined as follows:

(12) $$d_{k}=\frac{n(C_{k})}{n(R_{k})}=\frac{n(C_{k})}{l_{k}\cdot w_{k}}$$

where $n(C_{k})$ and $n(R_{k})$ denote the pixel number of LSR $C_{k}$ and its minimum enclosing rectangle $R_{k}$, $d_{k}$ is the aligned point density of LSR $C_{k}$. If the aligned point density $d_{k}$ exceeds the threshold $t_{d}$, the LSR represents an effective line feature, otherwise the LSR should be interpreted as several line features, that means it needs to be cut into several LSRs by the following methods:

*Method 1.* Reduce the tolerance of the region growing method to τ=π/16, mark all the pixels included in the LSR as “unlabeled” and repeat region growing on this pixel set, compute the aligned point density of the new LSR, if it still does not exceed threshold the $t_{d}$, try Method 2;

*Method 2.* Define the radius $r_{k}$ of LSR $C_{k}$ as the maximum distance between the seed point and all the other pixels in $C_{k}$, reduce $r_{k}$ to 80% of current value and remove all the outlier pixels from $C_{k}$, then repeat this procedure until the aligned point density $d_{k}$ exceeds threshold $t_{d}$. The threshold $t_{d}$ needs to be set by experience, if $t_{d}$ is set too large, the edges will be overcut, else if $t_{d}$ is set too small, the aforementioned curve and polyline problem cannot be solved; generally $t_{d}$ is set to 0.7.

**Figure 7:**
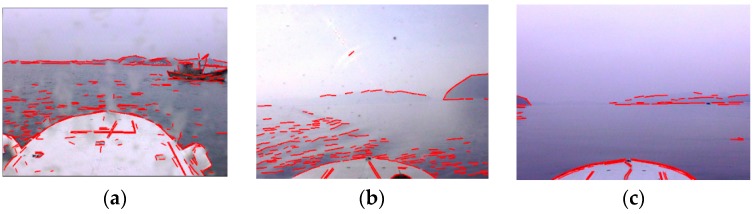
Line feature detection results of optical images in typical adverse weather conditions. (**a**–**c**) are optical images with extracted line features.

The computed line features of LSRs are shown in original optical images, as depicted in Figure 7. Note that the curve edges in images are approximately interpreted as several line segments due to improvement of line features. Consequently, the negative influence of various edges on SSL detection is effectively suppressed by improvement of line features, otherwise there will be huge error in computation of line features for SSL detection, when other edges accidentally intersect SSL with small angles.

## 4. Identification of SSL

**Figure 8:**
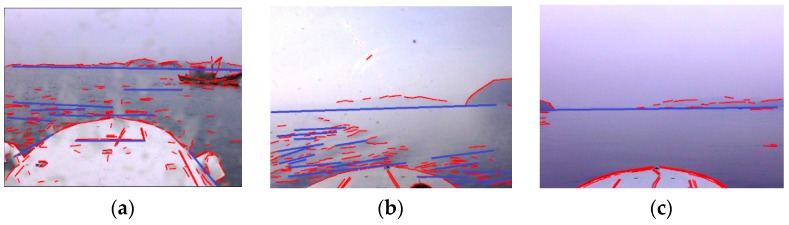
Line feature merging results of optical images in typical adverse weather conditions. (**a**–**c**) are optical images with improved line features.

If we observe the line feature detection results of optical images acquired under typical adverse weather condtions, it is easy to discover that there are gaps in the SSL, or even part of the SSL is missing due to the adverse effect of factors such as target position, illumination, rain, snow and fog. To achieve accurate identification of SSL, the line features of SSL need to be merged into an integral line feature first. Suppose that the line feature set detected from an optical image is denoted by $\left\{\psi_{k}\right\}$where $\psi_{k}$ is the unique parameter vector of a line feature:

(13) $$\psi_{k}={\left[x_{1k},y_{1k},x_{2k},y_{2k},{\bar{\theta }}_{k}\right]}^{T}$$

where $\left(x_{1k},y_{1k}\right)$ and $\left(x_{2k},y_{2k}\right)$ are coordinates of the start point and the end point of the line feature, ${\bar{\theta }}_{k}$ is the orientation of the line feature. Then the necessary and sufficient condition that two line features $\psi_{j}$ and $\psi_{k}$ belong to the same line segment is formulated as follows:

(14) $$\begin{array}{l} \left|{\bar{\theta }}_{j}-{\bar{\theta }}_{k}\right|<\delta \\ \left|\begin{array}{ccc} x_{1j} & y_{1j} & 1\\ x_{2j} & y_{2j} & 1\\ x_{1k} & y_{1k} & 1 \end{array}\right|<\lambda {\left\|\left(x_{1j},y_{1j})-(x_{2j},y_{2j}\right)\right\|}_{2}\\ \left|\begin{array}{ccc} x_{1j} & y_{1j} & 1\\ x_{2j} & y_{2j} & 1\\ x_{2k} & y_{2k} & 1 \end{array}\right|<\lambda {\left\|\left(x_{1j},y_{1j})-(x_{2j},y_{2j}\right)\right\|}_{2} \end{array}$$

where δ is the line feature orientation tolerance and δ=π/32, λ is the line feature offset tolerance and λ=2. When this condition is met, line features $\psi_{j}$ and $\psi_{k}$ are merged into a new line feature. To reduce the computational complexity of line feature merging, the line feature set $\left\{\psi_{k}\right\}$ is arranged by the order of orientation ${\bar{\theta }}_{k}$, so each time we only need to examine if two neighboring line features $\psi_{k}$ and $\psi_{k+1}$ satisfy the condition above. If there are $n_{\psi }$ line features in $\left\{\psi_{k}\right\}$, then the computational complexity is reduced from $O(n_{\psi }^{2})$ to $O(n_{\psi }\log n_{\psi })$. The experimental results of line feature merging are shown in Figure 8, where the blue line segments denote new line features, which are obtained by merging several line features that satisfy the condition above.

Note that besides the line feature denoted by SSL, there are other line features produced by wave glint, the USV hull, the target, mountains, *etc*. Therefore the SSL needs to be identified from among the line feature set according to region contrast, line segment length and orientation features. The region contrast $\eta_{k}$ of line feature $\psi_{k}$ is formulated as follows:

(15) $$\eta_{k}=\sum_{j\neq k}\exp\left(-\frac{{\left\|\left({\bar{x}}_{k},{\bar{y}}_{k})-({\bar{x}}_{j},{\bar{y}}_{j}\right)\right\|}_{2}^{2}}{\sigma_{\eta }^{2}}\right)n(C_{j})\left|\bar{S}(C_{k})-\bar{S}(C_{j})\right|$$

where $\bar{S}(C_{j})$ and $\bar{S}(C_{k})$ denote the mean gradient saliency of LSRs $C_{j}$ and $C_{k}$ corresponding to line features $\psi_{j}$ and $\psi_{k}$, respectively. $\left({\bar{x}}_{j},{\bar{y}}_{j}\right)$ and $\left({\bar{x}}_{k},{\bar{y}}_{k}\right)$ are the saliency centroids of $C_{j}$ and $C_{k}$, variance $\sigma_{\eta }$ controls the weighting strength of spatial distance between saliency centroids and in this paper $\sigma_{\eta }^{2}=0.64$ is used.

The likelihood $\mu_{k}$ of each line feature belonging to SSL can be calculated as follows:

(16) $$\mu_{k}=\exp\left(\frac{l_{k}}{l_{0}}-1\right)\frac{\eta_{k}}{\sum \eta_{j}}\cos{\bar{\theta }}_{k}$$

where $l_{k}$ and $l_{0}$ denote length of the line feature and the image diagonal, respectively. The line feature with the maximum likelihood $\mu_{k}$ will be selected as the SSL detection result.

## 5. Detection Accuracy Improvement

The Kalman filtering theory considers a processed signal as the system output under the effect of Gaussian white noise, and the relationship between input and output can be described by state space equations, thus the optimal state estimation can be recursively calculated by previous system state estimation and current measurement [17,18]. To solve the high dimensional nonlinear filtering problems, Haykin, *et al.* proposed a spherical-radial cubature rule to numerically compute multivariate moment integrals encountered in the nonlinear Bayesian filter, and this nonlinear filter, known as CKF, achieves higher accuracy and stability for state estimation of nonlinear system over conventional nonlinear filters [19,20]. There exist various interference factors like low contrast, low sharpness and noise in optical images from real marine environment, besides there are some approximations in SSL detection method, and those cause errors in SSL detection results.

**Figure 9:**
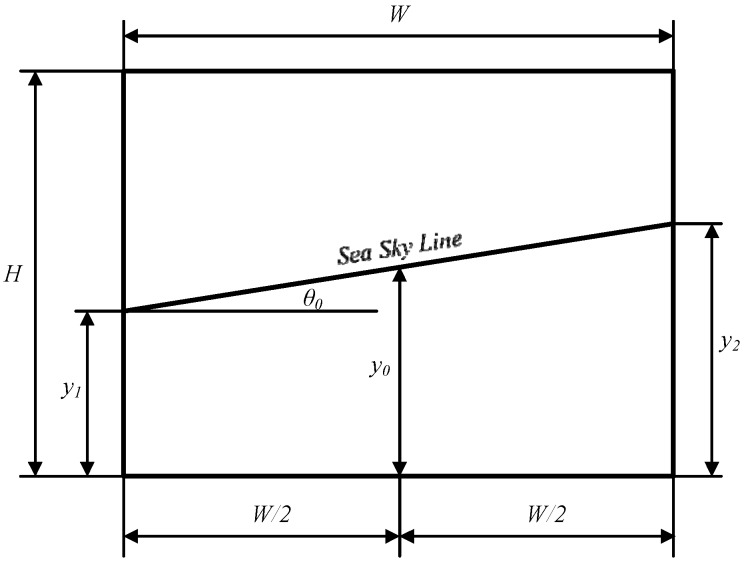
Geometric model of SSL detection.

To illustrate the noise distribution pattern in SSL detection results, we have mounted the optoelectronic imaging unit at the same height above the sea surface as the “XL” USV so that the camera is absolutely stationary without any impact of USV motion status. Optical images are acquired under different weather conditions and camera poses, and the SSL detection results are compared with the ground truth labeled by experts. The comparison verifies that the noise amplitude obeys a Gaussian distribution and its power spectral density is uniformly distributed, approximately. Thus we can use CKF to estimate the actual position of the SSL. The geometric model of SSL detection is shown in Figure 9, where *W* and *H* are the image width and height, $y_{1}$ and $y_{2}$ are vertical coordinates of points where the SSL intersects with the left and right image borders, $y_{0}$ is the vertical coordinate of the midpoint on the SSL, and $\theta_{0}$ is the orientation of the SSL.

The process equation for the SSL detection problem is formulated as follows:

(17) $${\hat{y}}_{k+1}=f\left({\hat{y}}_{k}\right)+v_{k}=\left[\begin{array}{cccccc} 1 & \Delta t & 0.5\cdot \Delta t^{2} & 0 & 0 & 0\\ 0 & 1 & \Delta t & 0 & 0 & 0\\ 0 & 0 & 1 & 0 & 0 & 0\\ 0 & 0 & 0 & 1 & \Delta t & 0.5\cdot \Delta t^{2}\\ 0 & 0 & 0 & 0 & 1 & \Delta t\\ 0 & 0 & 0 & 0 & 0 & 1 \end{array}\right]\cdot {\hat{y}}_{k}+v_{k}$$

where ${\hat{y}}_{k}$ is the system state at time k and ${\hat{y}}_{k}={\left[\begin{array}{cccccc} y_{1} & {\dot{y}}_{1} & {\ddot{y}}_{1} & y_{2} & {\dot{y}}_{2} & {\ddot{y}}_{2} \end{array}\right]}_{k}^{T}$, $v_{k}$ is Gaussian white noise with zero mean and covariance $Q_{k}$, Δt is the period for acquiring optical images.

The measurement equation is formulated as follows:

(18) $${\hat{z}}_{k+1}=h({\hat{y}}_{k+1})+w_{k+1}={\left[\begin{array}{c} \frac{y_{1}+y_{2}}{2}\\ \mathrm{atan}(\frac{y_{2}-y_{1}}{W}) \end{array}\right]}_{k+1}+w_{k+1}$$

where $w_{k+1}$ is Gaussian white noise with zero mean and covariance $R_{k+1}$. The cubature point set and the corresponding weights are set as follows [19]:

(19) $$\begin{array}{l} \varepsilon_{i}=\sqrt{\frac{m}{2}}{\left[1\right]}_{i}\\ \omega_{i}=\frac{1}{m},i=1,2,\cdots ,m=2n \end{array}$$

where ${\left[1\right]}_{i}$ is the i-th element of a complete fully symmetric set of points, n is state dimension and n=6 in this paper. The cubature Kalman filtering process is described as follows [20]:

### 5.1. Time Update

Factorize state covariance $P_{k|k}$ with Cholesky decomposition:

(20) $$P_{k|k}=S_{k|k}S_{k|k}^{T}$$

Evaluate the cubature points:

(21) $$Y_{i,k|k}=S_{k|k}\varepsilon_{i}+{\hat{y}}_{k|k},i=1,2,\cdots ,m=2n$$

Evaluate the propagated cubature points:

(22) $$Y_{i,k+1|k}^{*}=f(Y_{i,k|k})$$

Estimate the predicted state and error covariance:

(23) $$\begin{array}{l} {\hat{y}}_{k+1|k}=\frac{1}{m}\sum_{i=1}^{m}Y_{i,k+1|k}^{*}\\ P_{k+1|k}=\frac{1}{m}\sum_{i=1}^{m}Y_{i,k+1|k}^{*}Y_{i,k+1|k}^{*T}-{\hat{y}}_{k+1|k}{\hat{y}}_{k+1|k}^{T}+Q_{k} \end{array}$$

### 5.2. Measurement Update

Factorize predicted error covariance $P_{k+1|k}$ with Cholesky decomposition:

(24) $$P_{k+1|k}=S_{k+1|k}S_{k+1|k}^{T}$$

Evaluate the cubature points:

(25) $$Y_{i,k+1|k}=S_{k+1|k}\varepsilon_{i}+{\hat{y}}_{k+1|k},i=1,2,\cdots ,m=2n$$

Evaluate the propagated cubature points:

(26) $$Z_{i,k+1|k}=h(Y_{i,k+1|k})$$

Estimate the predicted measurement and error covariance:

(27) $$\begin{array}{l} {\hat{z}}_{k+1|k}=\frac{1}{m}\sum_{i=1}^{m}Z_{i,k+1|k}\\ P_{zz,k+1|k}=\frac{1}{m}\sum_{i=1}^{m}Z_{i,k+1|k}Z_{i,k+1|k}^{T}-{\hat{z}}_{k+1|k}{\hat{z}}_{k+1|k}^{T}+R_{k+1} \end{array}$$

Estimate the cross-covariance:

(28) $$P_{xz,k+1|k}=\frac{1}{m}\sum_{i=1}^{m}Y_{i,k+1|k}Z_{i,k+1|k}^{T}-{\hat{x}}_{k+1|k}{\hat{z}}_{k+1|k}^{T}$$

Estimate the Kalman gain:

(29) $$W_{k}=P_{xz,k+1|k}P_{zz,k+1|k}^{-1}$$

Estimate the updated state:

(30) $${\hat{y}}_{k+1|k+1}={\hat{y}}_{k+1|k}+W_{k}({\hat{z}}_{k+1}-{\hat{z}}_{k+1|k})$$

Update the state covariance:

(31) $$P_{k+1|k+1}=P_{k+1|k}-W_{k+1}P_{zz,k+1|k}W_{k+1}^{T}$$

### 5.3. Initial Conditions

The initial conditions of CKF for SSL detection are set as follows:

(32) $$\begin{array}{l} P_{0|0}=diag\left[\begin{array}{cccccc} 100.0 & 9.0 & 1.0 & 100.0 & 9.0 & 1.0 \end{array}\right]\\ {\hat{y}}_{0}={\left[\begin{array}{cccccc} 180.0 & 0.0 & 0.0 & 180.0 & 0.0 & 0.0 \end{array}\right]}^{T} \end{array}$$

The covariance matrices of process noise and measurement noise are set as follows:

(33) $$\begin{array}{l} Q_{k}=\left[\begin{array}{cc} \gamma & 0\\ 0 & \gamma \end{array}\right],\gamma =\left[\begin{array}{ccc} \Delta t^{5}/20 & \Delta t^{4}/8 & \Delta t^{3}/6\\ \Delta t^{4}/8 & \Delta t^{3}/3 & \Delta t^{2}/2\\ \Delta t^{3}/6 & \Delta t^{2}/2 & \Delta t \end{array}\right]\\ R_{k+1}=diag\left[\begin{array}{cc} 100.0 & 16.0 \end{array}\right] \end{array}$$

## 6. Experimental Results and Discussion

**Figure 10:**
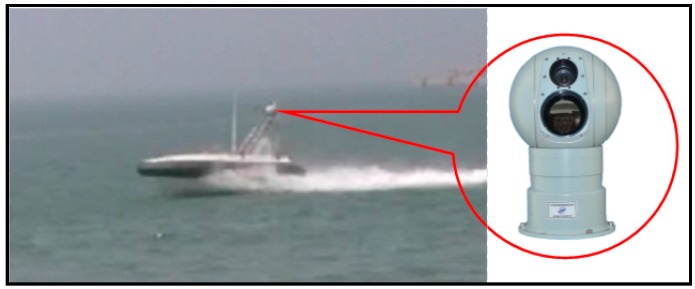
“XL” USV and the optoelectronic imaging unit.

To demonstrate the effectiveness and superiority of the proposed saliency based SSL detection method, the “XL” USV was used to acquire optical images of a marine environment in typical adverse weather like rainy weather, sunny weather with strong illumination, and foggy weather in the Penglai Sea area, Shandong Province, China, as shown in Figure 10. The exposure and focus of the optoelectronic imaging unit were set to auto mode, and the optical image resolution was set to 640 × 480. We have evaluated the proposed method on a benchmark dataset including 400 optical images and compared it against other state-of-the-art methods, including RANSAC line fitting [2], Hough transform [5], Radon transform [9] and shearlet transform [3]. The experimental environment was the C++ compiler of Microsoft Visual Studio 2012 on a Dual Core 2.5 GHz machine with 2 GB RAM. For the Hough transform and Radon transform, we used the authors’ implementations, while for RANSAC line fitting and shearlet transform, we implemented the algorithms in C++ since we failed to obtain the authors’ implementations.

**Figure 11:**
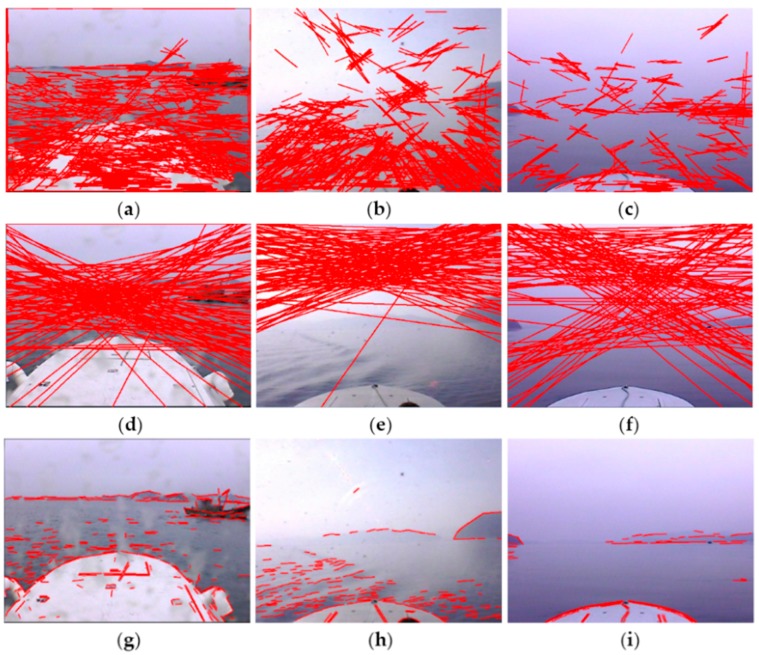
Line feature detection results of optical images: (**a**–**c**) are results of the Hough transform method; (**d**–**f**) are results of Radon transform method; (**g**–**i**) are results of the proposed method.

The proposed method is similar to Hough transform and Radon transform in line feature detection, therefore the performance of the three methods in line feature detection is contrasted first. Figure 11a–c shows the line feature detection results of the Hough transform. The basic principle of the Hough transform is to search for local peaks in Hough space to determine the line feature parameters, however, random edges caused by wave glints, illumination, mountains and cloud clusters accumulate in Hough space to form local peaks, and many mutually unrelated edges are connected in error to form false line features as a result, which causes great difficulty for the identification of the SSL. Figure 11d–f shows the line feature detection results of Radon transform. The Radon transform projects gradient maps into sinograms by line integrals, then the local peaks are searched to determine the line feature parameters, thus it is confronted with the same problem as the Hough transform, besides, the Radon transform can not determine the endpoints of line features. Figure 11g–i is the results obtained by the proposed method. The interference edges are obviously suppressed, and it is feasible to accurately identify the SSL from the detected line features. Therefore, the line feature detection performance of the proposed method is significantly superior to that of the Hough transform and Radon transform.

However, the SSL is usually weak or maybe even partly missing, so the interference edge points may randomly constitute lines which have many or even the most inliers, thus not only is the number of false alarmd of RANSAC line fitting rather high, but also the computational cost is enormously huge.

**Figure 12:**
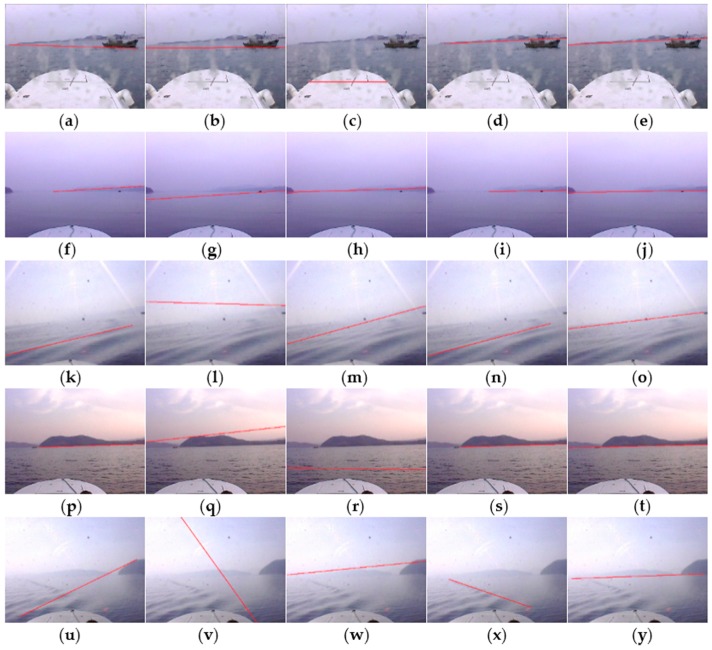
Comparison of SSL detection results. (**a**,**f**,**k**,**p**,**u**) are detection results of the Hough transformation method; (**b**,**g**,**l**,**q**,**v**) are detection results of the Radon transformation method; (**c**,**h**,**m**,**r**,**w**) are detection results of the RANSAC line fitting method; (**d**,**i**,**n**,**s**,**x**) are detection results of the shearlet transformation method; (**e**,**j**,**o**,**t**,**y**) are detection results of the proposed method.

Figure 12d,i,n,s,x are detection results of the shearlet transform. With the advantage of edge geometric features provided by the shearlet transform, the edge direction information is extracted and classified, but usually interference edges have better gradient orientation consistency than the relatively weak SSL, thus the detection accuracy of shearlet transform are not satisfactory, while the computational complexity is unacceptably huge.

Figure 12e,j,o,t,y shows the detection results of the proposed method. Obviously the SSL can be more accurately detected in the presence of various interfering factors, and the detection accuracy performance is superior to that of the other state-of-the-art methods. A detection result is considered to be accurate if it overlaps more than 50% of the real SSL. Based on this criterion the accuracy rates of the Hough transform, Radon transform, RANSAC line fitting, shearlet transform and the proposed method were statistically compared. Besides, the real-time requirement for application on USVs is considered and the average consumed time is also contrasted. As observed in Table 1, the accuracy rate and real-time performance of the proposed method significantly outperform other state-of-the-art methods. RANSAC line fitting gets the worst accuracy rate, and it takes a lot of time to process a single image due to random edge point selection and inlier verification. The shearlet transform achieves a better accuracy rate, but its computational complexity is huge and its real-time performance is the worst. Both the accuracy rate and the real-time performance of the Hough transform are similar to those of the Radon transform, but the Radon transform projected gradient maps into sinograms, while the Hough transform projects binary edge maps into Hough space, thus the accuracy rate of the Radon transform is slightly better but its average consumed time is a bit longer than that of the Hough transform.

**Figure 13:**
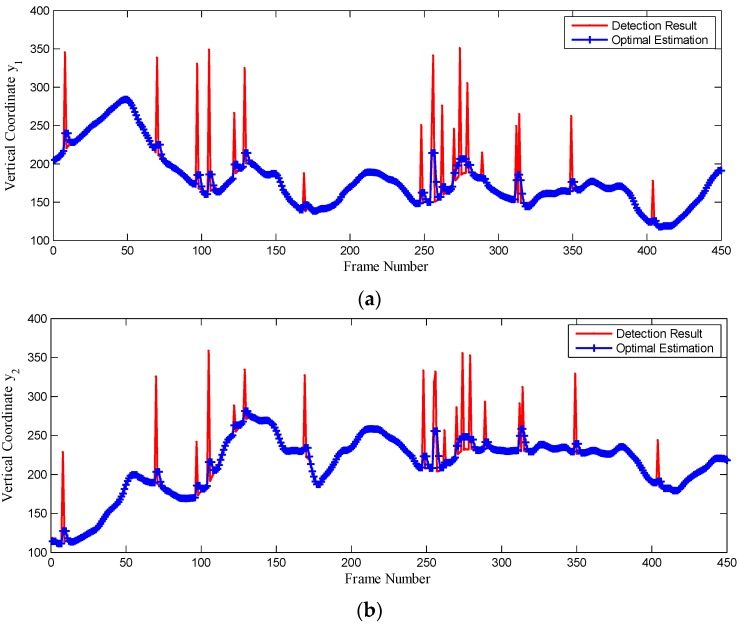
The SSL state comparison of detection results and state estimation by CKF. (**a**) are detection results and state estimation of vertical coordinate $y_{1}$; (**b**) are detection results and state estimation of vertical coordinate $y_{2}$.

In a real marine environment a sequence of optical images were continuously acquired by optoelectronic imaging unit with a sampling period Δt=80ms and processed by our proposed method online to detect the SSL. Taking 450 frames acquired during 36 s as an example, we compare the SSL detection results with state estimation by CKF, as depicted in Figure 13.

Generally, the vertical coordinates $y_{1}$ and $y_{2}$ should be continuously and smoothly changing with time k, yet there exist many peaks which represent abrupt changes in the SSL state caused by USV motion and various interference factors. Thus CKF is applied to estimate the optimal state of the SSL according to the previous state estimation and current measurement, which denotes the SSL detection result of the current image. As observed in Figure 13, the SSL state estimation by CKF is changing more smoothly with time, when it is accurately tracking the SSL state.

**Figure 14:**
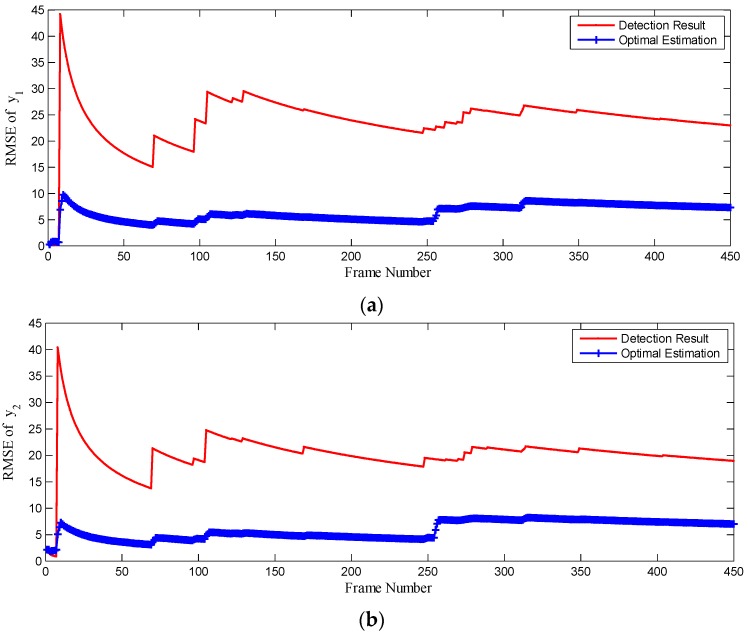
The RMSE comparison of detection results and state estimation by CKF. (**a**) is RMSE of detection results and state estimation of vertical coordinate $y_{1}$; (**b**) is RMSE of detection results and state estimation of vertical coordinate $y_{2}$.

To quantitatively evaluate the accuracy improvement by CKF, the SSL detection results and state estimation by CKF have been contrasted with the ground truth, which is the manually labeled SSL in the dataset by experts. The root mean square error (RMSE) at time k is defined as follows:

(34) $$RMSE(k)=\sqrt{\frac{1}{k}\sum_{i=0}^{k}{\left({\hat{y}}_{k}-{\hat{y}}_{k|k}\right)}^{2}}$$

where ${\hat{y}}_{k}$ is the ground truth at time k, and ${\hat{y}}_{k|k}$ is the detection result or state estimation by CKF at time k. The RMSE of detection results and state estimation by CKF is shown in Figure 14. After CKF applied to the proposed method, the RMSE of state estimation decreases by more than 50% and the accuracy of SSL detection is obviously improved.

The proposed method has been used on the “XL” USV to accelerate target searching by reducing the search area and computational complexity. The sea trial results show that the search time for a single target decreases by more than 82% with knowledge of the SSL location. Future research work will be concentrated on accurate noise modeling with compensation of USV motion status so that nonlinear Bayesian filtering method could separate the noise to further improve the accuracy and stability of SSL detection method. The proposed method could also be used for horizon detection of monochromatic images such as infrared images or spectrum images.

## 7. Conclusions

Through the computation of gradient saliency, the line features of the SSL in optical images acquired in typical adverse weather can be effectively enhanced, while other interference factors are relatively suppressed. The region growing method on gradient orientation can accurately extract line features which have good gradient orientation consistency, meanwhile avoiding the problems in other line feature detection methods like the Hough transform and Radon transform where mutually unrelated edges often get connected by mistake to form false line features. Experimental results from the “XL” USV in typical adverse weather demonstrate that the proposed method is significantly superior to other state-of-the-art methods in terms of accuracy rate and real-time performance, and its accuracy and stability has been further improved by CKF.

## Figures and Tables

**Table 1:** SSL detection result comparison of different methods on the benchmark dataset.

Measure	Hough Transform	Radon Transform	Ransac Line Fitting	Shearlet Transform	The Proposed Method
Accuracy rate	76.8%	79.0%	67.3%	84.3%	94.8%
Average consumed time	167 ms	185 ms	1354 ms	5629 ms	52 ms
